# Prevalence of Abnormalities and Normal Variants in the Adolescent Knee on MRI in a Population-Based Cohort of 3800 Knees

**DOI:** 10.1177/03635465241277162

**Published:** 2024-09-15

**Authors:** Laura A.M. Kemmeren, Edwin H.G. Oei, Marienke van Middelkoop, Denise Eygendaal, Tom M. Piscaer

**Affiliations:** † Department of Orthopaedics and Sports Medicine, Erasmus Medical Center, Rotterdam, the Netherlands; ‡ Department of Radiology and Nuclear Medicine, Erasmus Medical Center, Rotterdam, the Netherlands; § Department of General Practice, Erasmus Medical Center, Rotterdam, the Netherlands; Investigation performed at Erasmus Medical Center, Rotterdam, the Netherlands

**Keywords:** knee, articular cartilage, meniscus, osteochondritis dissecans, patella, patellar tendon, pediatric sports medicine, aging athlete, epidemiology, anatomy, magnetic resonance imaging

## Abstract

**Background:**

Many adolescents experience knee pain, and only some undergo detailed imaging. In this population, the prevalence of abnormalities and normal variants on magnetic resonance imaging (MRI) scans is unknown.

**Purpose:**

To investigate the prevalence of abnormalities and normal variants of the knee on MRI scans and their relationship with participant characteristics in the general young adolescent population.

**Study Design:**

Cross-sectional study; Level of evidence, 3.

**Methods:**

This study was part of an open population-based cohort study that focuses on health, growth, and development from fetal life until adulthood. Between 2017 and 2020, adolescents aged 12 to 15 years underwent MRI of both knees. These MRI scans were assessed in a standardized way for abnormalities and normal variants to determine their prevalence. Logistic regression was used to analyze the presence of abnormalities and normal variants in relation to sex, height, weight, body mass index–standard deviation (BMI-SD), and ethnicity.

**Results:**

A total of 1910 participants (median age, 13.5 years; interquartile range, 13.4-13.7 years; 52% girls) were included in this study. Of them, 370 (19.4%) participants had at least 1 abnormality or normal variant. Bone marrow edema around the knee was the most prevalent finding, affecting 140 (7.3%) participants. In 107 (5.6%) participants, nonossifying fibromas were found. A total of 43 (2.3%) participants had characteristics of Osgood-Schlatter disease, 16 (0.8%) showed characteristics of Sinding-Larsen-Johansson syndrome, and osteochondritis dissecans was found in 13 (0.7%) participants. Variants such as discoid menisci were found in 40 (2.1%) participants and a bipartite patella in 21 (1.1%) participants. There were multiple associations between abnormalities or variants and participant characteristics, including bone marrow edema being more often present in boys (odds ratio [OR], 2.44; 95% CI, 1.69-3.52) and those with a lower BMI-SD (OR, 0.85; 95% CI, 0.73-0.98). Osgood-Schlatter and osteochondritis dissecans were more often present in boys (OR, 4.21 [95% CI, 2.01-8.85] and OR, 13.18 [95% CI, 1.71-101.58], respectively). Discoid menisci were associated with a non-Western ethnicity (OR, 2.06; 95% CI, 1.07-3.96) and higher BMI-SD (OR, 2.34; 95% CI, 1.76-3.11).

**Conclusion:**

Abnormalities and normal variants on MRI scans of the knees are common in adolescents. Physicians who are involved in the treatment of adolescents with knee pain need to be aware of this prevalence so that these children will not be overtreated or misdiagnosed.

Adolescence constitutes a critical period for growth and development, during which the musculoskeletal system undergoes significant changes. The knee in particular is a complex joint that undergoes many modifications in this period, potentially resulting in alterations in joint alignment, shape, and laxity.^26,29,34^ Knee pain is very common in children as well as adults and carries a significant global burden.^1^ The prevalence of knee pain is reported to be between 18.5% and 30.5% in adolescents.^9,31,32^ Children who experience knee pain have a higher risk of recurrent knee pain and subsequent complications, such as osteoarthritis, later in life.^12,31^ This underscores the importance of an enhanced understanding about the changes in the knee in this period of life.

Magnetic resonance imaging (MRI) is a powerful tool that can detect and visualize abnormalities and normal variants at an early stage. Previous studies have shown that abnormalities and normal variants of the knee on MRI scans are very common in symptomatic as well as asymptomatic adults, with a prevalence of up to 97% of MRI scans showing at least 1 abnormality or normal variant. Most of these studies concerned radiological features of osteoarthritis in adults or injuries in small groups of athletes.^8,13,20^ The prevalence of abnormalities and normal variants in the general population of adolescents, unselected by symptoms, disease, or risk factors, is currently unknown.

This study aimed to establish the prevalence of abnormalities and variants of the knee in the general adolescent population and their relationship with different participant characteristics. This was done by assessing >1900 knee MRI scans for abnormalities and normal variants.

## Methods

### Study Population

This study used data from the Generation R cohort, a population-based prospective cohort study focusing on health, growth, and development from fetal life until adulthood. Mothers due to deliver between April 2002 and January 2006 were invited to participate in this study. Measurements on them and their children were performed during preschool age and at 6, 10, 13, and 17 years of age. Written informed assent and consent were obtained from all children and their parents, respectively, and the study was approved by the Medical Ethics Committee of Erasmus Medical Center, Rotterdam, the Netherlands. More information on the study protocol and other measurements can be found in the design and cohort paper.^21^

For the current study, we used data of participants at the 13-year visit. At this age, some of these children received a full-body MRI study, including detailed imaging of their knees. This specific knee MRI study was not performed at earlier time points. Not all children who were in the follow-up at 13 years of age were invited for the knee MRI study because of capacity constraints. Children from the cohort were invited randomly to participate in the full-body MRI study, and they received high-quality MRI scans of the knees, hips, and/or brain. Which participant received which scan was based on contraindications for any of these scans (eg, braces for the brain MRI) and whether they had already received an MRI scan of the hips or brain at the 9-year follow-up. Children were included in this study, irrespective of whether they had knee pain. Children were not free to choose which body part they wanted an MRI study performed for. Furthermore, the MRI needed to be of sufficient image quality, meaning there were no major artifacts. Data collection took place between 2017 and 2020.

### Measurements

Data on age, height, weight, and body mass index–standard deviation (BMI-SD) at their visit; sex; and ethnicity were available for all children. Height and weight were used to calculate the BMI-SD scores according to the Dutch reference growth curves.^10^ BMI-SD scores were subdivided into different categories according to the cutoff values defined by Cole and Lobstein.^4^ The 3 categories for underweight were combined, resulting in the following 4 categories: underweight, normal weight, overweight, and obesity. All researchers were blinded to all participant characteristics during the assessment of the MRI scans.

### Magnetic Resonance Imaging

All MRI scans were performed using a dedicated 3-T MRI scanner (Discovery MR750w; GE Healthcare). Children were scanned according to standard imaging and positioning protocols for the Generation R study.^21^ The participants’ knees were scanned using a GRASS (gradient recalled acquisition in the steady-state) 3-dimensional sequence and a LAVA (liver acquisition with volume acceleration) flex sequence, providing in-phase and out-phase imaging.^25^ The slice thickness was 0.7 mm for the GRASS 3-dimensional sequence and 1.00 mm for the LAVA flex sequence. For both sequences, the in-plane (reconstructed) voxel size was 0.7 × 0.7 mm^2^.

Several abnormalities and well-described anatomic variants were evaluated and scored systematically on the MRI scans. This list was determined via expert opinion and existing literature and was based on conditions that were likely to occur in this age group and could be well visualized on the acquired MRI scans. These items included the presence of bone marrow edema, discoid menisci (graded as complete or incomplete),^18^ bipartite patellae (graded as type 1, inferior; type 2, lateral; or type 3, superolateral),^22^ and signs of Osgood-Schlatter disease (defined as the radiological presence of edema of the tibial tuberosity, distal patellar tendon, and adjacent fat and/or the presence of an ossicle at the tibial tuberosity).^11,24^ Furthermore, characteristics of Sinding-Larsen-Johansson syndrome (defined as edema in the proximal patellar tendon, inferior pole of the patella, and adjacent fat of the Hoffa fat pad or the presence of an ossicle at the inferior pole of the patella),^23^ the presence of osteochondritis dissecans, the presence of nonossifying fibromas, focal periphyseal edema, and general edema of the Hoffa fat pad were assessed. Other bony and chondral abnormalities found on the MRI scans were also documented.

MRI scans were assessed by a musculoskeletal radiologist at the time they were performed for major incidental findings that needed follow-up by a medical specialist. In this case, the participants and their parents were contacted. Participants were not informed about any abnormality that was found. For this study, all MRI scans were assessed by L.A.M.K. after training by E.H.G.O., a senior musculoskeletal radiologist, and T.M.P., a senior orthopaedic surgeon specialized in knee surgery. All abnormalities that were found were reevaluated by T.M.P. When in doubt, E.H.G.O. was consulted, and agreement was reached between the 3 authors. A random sample of 50 MRI scans was taken and evaluated by both L.A.M.K. and T.M.P. to assess interobserver reliability. The prevalence-adjusted bias-adjusted kappa was 0.84 for finding any abnormality, indicating very good interrater agreement.^5^

### Statistical Analysis

Descriptive statistics were used to describe participant characteristics, as well as any abnormalities and normal variants found on MRI scans. Medians and interquartile ranges (IQRs) were calculated when data were not normally distributed. Logistic regression was used to analyze the presence of abnormalities in relation to sex, height, weight, BMI-SD, and ethnicity. Results are reported as odds ratios (OR) and their 95% CIs. All analyses were performed using SPSS software (Version 28.0; IBM Corp).

## Results

**Figure 1.**
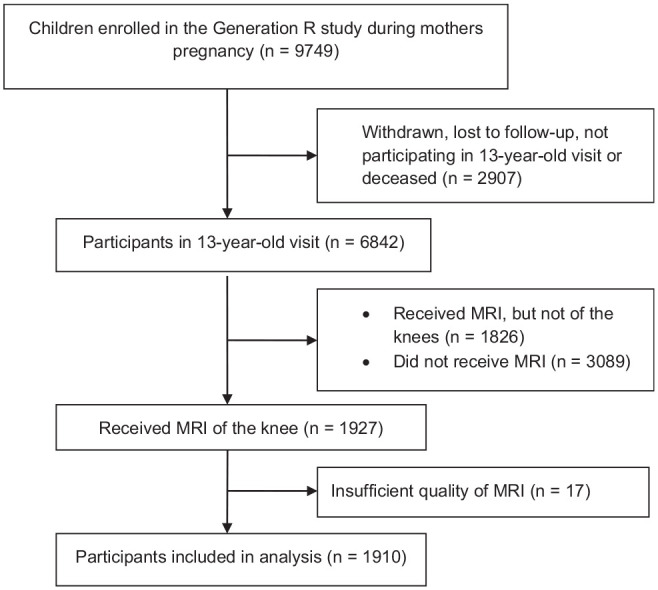
Flow diagram for the study sample selection. MRI, magnetic resonance imaging.

The process of establishing the study sample is displayed in Figure 1. Of the 9749 live-born children at the start of the Generation R study, 6842 participated in the 13-year visit. Not every child received MRI and not every child received MRI of the knees. A total of 1927 participants underwent detailed MRI of both knees. Of these scans, 1910 (3820 knees) were of sufficient quality and were therefore included in this study. Participants who underwent MRI were more often female than those who did not. There were no other statistically significant differences between these groups (see the Appendix, available in the online version of this article). The median age of the participants was 13.5 years (IQR, 13.4-13.7 years, range 12-15 years). Patient characteristics are presented in Table 1.

**Table 1:** Characteristics of Study Participants (N = 1910)^*a*^

	Value
Female sex	994 (52.0)
Age, y	13.5 (13.4 to 13.7)
Ethnicity	
Dutch	1150 (61.4)
Other Western	161 (8.6)
Non-Western	561 (30.0)
Height, cm	164.1 (158.9 to 169.6)
Weight, kg	52.2 (46.0 to 60.0)
BMI-SD	0.2 (–0.6 to 1.0)
Weight category	
Underweight	217 (11.4)
Normal weight	1390 (72.9)
Overweight	247 (12.9)
Obese	54 (2.8)

a Data are given as median (interquartile range) for continuous variables and absolute number (percentage) for categorical variables. Data were missing for the following: ethnicity (n = 38), height (n = 1), weight (n = 1), BMI-SD (n = 1), and weight category (n = 2). BMI-SD, body mass index–standard deviation.

### Prevalence of Abnormalities and Normal Variants

A total of 370 (19.4%) participants had at least 1 abnormality or normal variant (Table 2). Bone marrow edema was the most prevalent abnormality, observed in 140 (7.3%) participants. In 107 (5.6%) participants, nonossifying fibromas were found. In total, 43 (2.3%) participants had characteristics of Osgood-Schlatter disease, and 16 (0.8%) showed features of Sinding-Larsen-Johansson syndrome. Osteochondritis dissecans was found in 13 (0.7%) participants; discoid menisci, in 40 (2.1%) participants; and a bipartite patella, in 21 (1.1%) participants.

**Table 2:** Prevalence of Abnormalities Found on MRI^*a*^

	Unilateral	Bilateral	Total Participants	Prevalence, %
Bone marrow edema			140	7.3
Medial femoral condyle	63	14	77	4.0
Lateral femoral condyle	12	1	13	0.7
Medial tibial plateau	11	2	13	0.7
Lateral tibial plateau	15	2	17	0.9
Femur	1	0	1	0.1
Tibia	11	2	13	0.7
Fibula	6	1	7	0.4
Patella	23	1	24	1.3
Features of Osgood-Schlatter disease			43	2.3
Edema of tibial tuberosity	24	5	29	1.5
Edema of distal patellar tendon	23	6	29	1.5
Edema of adjacent fat	12	1	13	0.7
Ossicle of tibial tuberosity	18	0	18	0.9
Features of Sinding-Larsen-Johansson syndrome			16	0.8
Edema of proximal patellar tendon	9	1	10	0.5
Edema of inferior pole patella	7	1	8	0.4
Edema of adjacent fat	3	0	3	0.2
Ossicle of inferior pole patella	4	1	5	0.3
Osteochondritis dissecans	11	2	13	0.7
Discoid meniscus			40	2.1
Type 1 (complete)	5	9	14	0.7
Type 2 (incomplete)	13	13	26	1.4
Bipartite patella			21	1.1
Type 1 (inferior pole)	1	1	2	0.1
Type 2 (lateral margin)	5	0	5	0.3
Type 3 (superolateral pole)	12	2	14	0.7
Focal periphyseal edema			8	0.4
Femur	5	0	5	0.3
Tibia	2	1	3	0.2
Fibula	0	0	0	0.0
Nonossifying fibroma			107	5.6
Femur	56	3	59	3.1
Tibia	48	2	50	2.6
Fibula	3	0	3	0.2
Edema of Hoffa fat pad	4	7	11	0.6
Infrapatellar edema/bursitis deep bursa	3	0	3	0.2
Dorsal defect of patella	6	2	8	0.4
Osteochondral defect of medial facet of patella	1	0	1	0.1
Subchondral defect of patella	3	0	3	0.2
Osteochondroma	1	0	1	0.1
Unspecified chondroid lesion of metaphysis	1	0	1	0.1
Unspecified chondroid lesion of epiphysis	2	1	3	0.2
Epiphysis cyst	3	0	3	0.2
Ganglion cyst in Hoffa fat pad	1	0	1	0.1

a Data are given as absolute numbers unless otherwise indicated. When the number of children affected, given at the top of the row, is lower than the total number of affected children in the corresponding rows below, this is because multiple characteristics occurred in the same participant. MRI, magnetic resonance imaging.

### Associations Between Abnormalities and Normal Variants and Participant Characteristics

Associations between abnormalities and normal variants and participant characteristics are presented in Table 3. The participants with bone marrow edema were more often boys (OR, 2.44; 95% CI, 1.69-3.52). Signs of Osgood-Schlatter disease and osteochondritis dissecans were also more often seen in boys (OR, 4.21 [95% CI, 2.01-8.85] and OR, 13.18 [95% CI, 1.71-101.58], respectively). Those with bone marrow edema were also more likely to have a lower BMI-SD (OR, 0.85; 95% CI, 0.73-0.98). Discoid menisci were associated with a non-Western ethnicity (OR, 2.06; 95% CI, 1.07-3.96), being overweight or obese (OR, 6.44; 95% CI, 3.32-12.50), greater weight (OR, 1.05; 95% CI, 1.03-1.07), lower height (OR, 0.94; 95% CI, 0.90-0.98), and higher BMI-SD (OR, 2.34; 95% CI, 1.76-3.11).

**Table 3:** Associations Between Abnormalities and Normal Variants and Participant Characteristics^*a*^

	Bone marrow edema	Signs of Osgood- Schlatter Disease	Signs of Sinding- Larsen-Johansson Syndrome	Osteochondritis Dissecans	Discoid Meniscus	Bipartite Patella	Nonossifying Fibroma
Sex							
Female	1	1	1	1	1	1	1
Male	**2.44 (1.69-3.52)**	**4.21 (2.01-8.85)**	1.82 (0.66-5.02)	**13.18 (1.71-101.58)**	0.82 (0.43-1.59)	2.56 (0.98-6.68)	1.25 (0.85-1.85)
Ethnicity							
Dutch	1	1	1	1	1	1	1
Other Western	0.66 (0.33-1.33)	0.65 (0.15-2.77)	0.00	0.79 (0.10-6.30)	0.00	0.65 (0.08-5.05)	1.20 (0.60-2.40)
Non-Western	0.71 (0.48-1.06)	1.68 (0.90-3.13)	1.93 (0.72-5.17)	0.64 (0.17-2.37)	**2.06 (1.07-3.96)**	1.40 (0.56-3.50)	1.20 (0.78-1.82)
Weight category							
Underweight	1.30 (0.78-2.14)	0.19 (0.03-1.40)	0.49 (0.06-3.77)	1.42 (0.31-6.65)	0.00	1.99 (0.64-6.16)	0.92 (0.48-1.77)
Normal weight	1	1	1	1	1	1	1
Overweight/obese	0.86 (0.52-1.43)	1.27 (0.60-2.68)	0.71 (0.16-3.16)	1.03 (0.22-4.78)	**6.44 (3.32-12.50)**	1.07 (0.30-3.77)	1.23 (0.74-2.05)
Weight	0.99 (0.97-1.00)	1.01 (0.99-1.04)	1.01 (0.97-1.05)	1.00 (0.95-1.05)	**1.05 (1.03-1.07)**	0.97 (0.92-1.02)	1.01 (0.99-1.02)
Height	1.00 (0.98-1.03)	1.02 (0.98-1.06)	1.02 (0.96-1.09)	1.06 (0.99-1.13)	**0.94 (0.90-0.98)**	0.96 (0.91-1.02)	1.00 (0.98-1.02)
BMI-SD score	**0.85 (0.73-0.98)**	1.20 (0.94-1.55)	1.08 (0.72-1.63)	0.87 (0.55-1.38)	**2.34 (1.76-3.11)**	0.83 (0.57-1.21)	1.10 (0.93-1.29)

a Data are given as OR (95% CI). When the OR is 0.00, no participants in that group had that abnormality and the OR was not calculated. Statistically significant ORs are given in bold. Data were missing for the following: ethnicity (n = 38), height (n = 1), weight (n = 1), BMI-SD score (n = 1), and weight category (n = 2). BMI-SD, body mass index–standard deviation; OR, odds ratio.

## Discussion

In this study, we aimed to investigate the prevalence of abnormalities and variants in the knees of adolescents in a general population using MRI. To our knowledge, such a study has not been conducted before, particularly not on such a large scale or in a sample unselected by symptoms, disease, or risk factors. The findings revealed that almost 1 in 5 participants had at least 1 abnormality or normal variant. Furthermore, we found that the presence of Osgood-Schlatter, osteochondritis dissecans, and bone marrow edema was associated with being male. We also found that discoid menisci were associated with a non-Western ethnicity, higher weight, BMI, and lower height.

It is important to note that although the prevalence of abnormalities and variants encountered on MRI scans was high, this does not always mean that those abnormalities have clinical consequences at the moment of the MRI study or in the future. Many abnormalities found are likely asymptomatic, such as bipartite patellae, or transient, such as bone marrow edema. Other findings may become symptomatic in the future in some individuals, such as discoid menisci. Because abnormalities were found so frequently, many are likely not symptomatic and can be considered incidental findings or normal variants in this age group.

Bone marrow edema was the most prevalent abnormality identified, affecting 7.3% of participants. Several studies have suggested that in small groups of athletes, the prevalence of bone marrow edema is estimated to be between 14% and 63%.^27,33,37^ Interestingly, boys showed a higher prevalence of knee bone marrow edema in this study. A possible explanation might be that they are more involved in collision or impact sports and experience more traumatic incidents because of this.^6^ A lower BMI-SD was also associated with more bone marrow edema, which also might be explained by children with a lower BMI-SD being more active in sports.^2^ Overall, it can be concluded that bone marrow edema is an abnormality that is very often found in the general population and even more often in active adolescents and can likely be considered a normal variant in this age group.

Nonossifying fibromas, which are reportedly common in children, were found in 5.6% of the participants in our study. One study reported a prevalence of 9.3% of participants having at least 1 nonossifying fibroma in a cohort with a mean age of 11 years that had undergone knee radiography.^7^ Our study's lower prevalence at a median age of 13.5 years supports the hypothesis that nonossifying fibromas disappear with age. However, a prevalence of nonossifying fibromas has not yet been documented in adults, which further supports this hypothesis.

The prevalence of Osgood-Schlatter disease was 2.3%, which is lower than the prevalence of around 10% reported in 12- to 15-year-old athletes.^14,28^ This difference can be explained by the strong association between physical activity and the presence of Osgood-Schlatter disease.^30^ We found that Osgood-Schlatter disease occurred more often in boys (OR, 4.21; 95% CI, 2.01-8.85), a relationship that has been established previously.^36^ However, this difference could be explained by the differences in skeletal maturation between boys and girls at this age. Osgood-Schlatter disease is most prevalent between ages 12 and 15 years for boys and 8 and 12 years for girls. Because the median age in this study was 13.5 years, girls may have outgrown the disease at the time of the MRI.

We found a relatively high prevalence of 0.7% of participants having osteochondritis dissecans as opposed to 0.095% in a previous study.^17^ Osteochondritis dissecans is a condition that has been underdiagnosed in the past, as plain radiography, which is the imaging modality primarily used in previous studies, often does not visualize osteochondritis dissecans properly. Furthermore, the early stages of this condition might be asymptomatic or cause only vague symptoms, leading to underdiagnosis in clinical practice. Another relatively rare condition is the dorsal defect of the patella, likely caused by a delayed ossification process of the superolateral quadrant of the patella. We found 8 cases of this abnormality, of which 2 were bilateral cases, resulting in a prevalence of 0.4%. This aligns with findings of 0.4% and 1.0% in previous studies on adolescents.^15,35^

Regarding discoid menisci, the prevalence of 2.1% observed in this study was on the lower end of the estimated range of 3% to 5% in the Western world. Participants with a non-Western ethnicity showed a higher frequency of discoid menisci (OR, 2.06; 95% CI, 1.07-3.96), which aligns with previous literature.^16,19^ Interestingly, higher weight and BMI were associated with a higher prevalence of this condition (OR, 1.05 [95% CI, 1.03-1.07] and OR, 2.34 [95% CI, 1.76-3.11], respectively), a relationship not previously described. This finding may be coincidental given the low prevalence overall.

### Strengths and Limitations

A significant strength of this study is the large sample size. No study has been performed before in which almost 2000 adolescents had their knees analyzed using MRI. Moreover, this study is based on an unselected population and therefore is representative of the general population. In this study, we systematically analyzed and described all abnormalities and normal variants that were found. Furthermore, we gained insights into multiple abnormalities and normal variants of the knee that can be found at this age and their relationship with sex, ethnicity, height, and weight.

Another strength is that the MRI scans were conducted using a 3-T MRI scanner, as opposed to a 1.5- or 2-T MRI scanner, which has been used in previous studies, allowing for significantly higher MR image quality and more accurate identification of abnormalities and variants, even in their early stages.^3^ On the other hand, we did not use the standard T1- and T2-weighted sequences commonly used in clinical protocols given the specific research purposes of the MRI scans obtained in the Generation R study. Although we found the applied water excitation GRASS sequence to be highly fluid sensitive, this may have led to under- or overrepresentation of certain radiological features, as they might be more or less clear on the sequences that were available to us, as opposed to T1- and T2-weighted sequences.

Another limitation of this study is the cross-sectional design: MRI scans were obtained at a single time point. Analysis of the same participants later in time will provide insights into the progression or regression of the identified abnormalities. The Generation R study is an ongoing study with multiple prospected follow-up time points in the future, beginning with a follow-up MRI at the age of 17 years. Furthermore, this study solely focused on findings on MRI scans and did not assess the clinical significance or symptoms associated with these abnormalities. It should be noted that abnormalities and variants found do not always cause pain or have clinical consequences and should therefore always be correlated to the examination, as many complaints can be innocuous and self-limiting.

## Conclusion

Abnormalities and normal variants of the knee are common in adolescents. We found that bone marrow edema is common in adolescents, especially in boys and those with a lower BMI, and that osteochondritis dissecans is more prevalent than previously thought. We also found that Osgood-Schlatter disease and osteochondritis dissecans are more common in boys and discoid menisci are more often found in adolescents with a non-Western ethnicity and a higher BMI-SD. Some of the abnormalities that are found can have consequences for a child's future, whereas others can be considered incidental findings. More research is needed to correlate the abnormalities found to pain and other symptoms to determine their clinical relevance.

## Supplemental Material

sj-pdf-1-ajs-10.1177_03635465241277162 – Supplemental material for Prevalence of Abnormalities and Normal Variants in the Adolescent Knee on MRI in a Population-Based Cohort of 3800 Knees Supplemental material, sj-pdf-1-ajs-10.1177_03635465241277162 for Prevalence of Abnormalities and Normal Variants in the Adolescent Knee on MRI in a Population-Based Cohort of 3800 Knees by Laura A.M. Kemmeren, Edwin H.G. Oei, Marienke van Middelkoop, Denise Eygendaal and Tom M. Piscaer in The American Journal of Sports Medicine
