# Supplementary material for: Prevalence of Abnormalities and Normal Variants in the Adolescent Knee on MRI in a Population-Based Cohort of 3800 Knees
Source: Am J Sports Med. 2024 Sep 15;52(12):3039–45. doi: 10.1177/03635465241277162 (PMC11529136; doi:10.1177/03635465241277162)
Supplement: sj-pdf-1-ajs-10.1177_03635465241277162 – Supplemental material for Prevalence of Abnormalities and Normal Variants in the Adolescent Knee on MRI in a Population-Based Cohort of 3800 Knees [file sj-pdf-1-ajs-10.1177_03635465241277162.pdf]

Prevalence of abnormalities of and normal variants in the adolescent knee on MRI in a population-based cohort of 3800 knees

Appendix

Demographics of the included participants in comparison to the participants of the Generation R study that were not included in this analysis[a]

	**Included participants (n=1910)**	**Excluded participants (n=4932)**	**p-value**
Sex, boy	916 (48.0)	2523 (51.2)	**0.018**
Age, years	13.5 (13.4-13.7)	13.5 (13.4-13.7)	0.62
Ethnicity			0.06
Dutch	1150 (61.4)	2836 (59.5)	
Other Western	161 (8.6)	367 (7.7)	
Non-Western	561 (30.0)	1562 (32.8)	
Height, cm	164.1 (159.2-169.5)	164.3 (158.7-169.7)	0.59
Weight, kg	52.2 (45.9-59.6)	52.2 (46.1-60.2)	0.17
BMI	19.1 (17.5-21.4)	19.2 (17.5-21.4)	0.09
BMI-SD	0.2 (-0.6-1.0)	0.3 (-0.5-1.0)	0.10
Weight category			0.07
Underweight	217 (11.4)	294 (9.8)	
Normal weight	1390 (72.9)	2210 (73.4)	
Overweight	247 (12.9)	388 (12.9)	
Obese	54 (2.8)	118 (3.9)	

[a] Values are given as median (interquartile range) for continuous variables or absolute number (percentage) for categorical variables. Missings were 205 for ethnicity and 1924 for weight, height, BMI and weight category. BMI = body mass index, BMI-SD = body mass index-standard deviation.
